# The lack of knowledge on acute stroke in Brazil: A cross-sectional study with children, adolescents, and adults from public schools

**DOI:** 10.1016/j.clinsp.2022.100052

**Published:** 2022-06-28

**Authors:** Marcelo Calderaro, Igor C. Salles, Gabriela B. Gouvêa, Vinícius S. Monteiro, Antonio P. Mansur, Henrique N.I. Shinohara, Priscila Aikawa, Iracema I.K. Umeda, Federico Semeraro, Maria José C. Carmona, Bernd W. Böttiger, Naomi K. Nakagawa

**Affiliations:** a KIDS SAVE LIVES Brazil, Education, Assessment and Intervention in Cardiopulmonary Group, Faculdade de Medicina da Universidade de São Paulo, São Paulo, SP, Brazil; b Neurology Department, Faculdade de Medicina da Universidade de São Paulo, São Paulo, SP, Brazil; c Cardiology Division, Instituto do Coração (InCor), Faculdade de Medicina da Universidade de São Paulo, São Paulo, SP, Brazil; d Physiology Department, Faculdade de Medicina da Universidade Federal do Rio Grande, Rio Grande, RS, Brazil; e Physiotherapy Department, Faculdade de Medicina da Universidade de São Paulo, São Paulo, SP, Brazil; f Anaesthesia and Intensive Care Department, Ospedale Maggiore, Bologna, Italy; g Anesthesiology Discipline, Faculdade de Medicina da Universidade de São Paulo, São Paulo, SP, Brazil; h Anaesthesiology and Intensive Care Medicine Department, University Hospital and Medical Faculty of the University Cologne, Cologne, Germany

**Keywords:** Stroke, Adolescents, Children, FAST, Risk factors, Signs and symptoms, WSS, Warning Signs and Symptoms (WSS), ARF, Associated Risk Factors, KSLB, KIDS SAVE LIVES BRAZIL, EMS, Emergency Medical Service

## Abstract

**Objective:**

Stroke is an important cause of disability and death in adults worldwide. However, it is preventable in most cases and treatable as long as patients recognize it and reach capable medical facilities in time. This community-based study investigated students' stroke knowledge, Emergency Medical Services (EMS) activation, associated risk factors, warning signs and symptoms, and prior experience from different educational levels in the KIDS SAVE LIVES BRAZIL project.

**Methods:**

The authors conducted the survey with a structured questionnaire in 2019‒2020.

**Results:**

Students from the elementary-school (*n* = 1187, ∼13 y.o., prior experience: 14%, 51% women), high-school (*n* = 806, ∼17 y.o., prior experience: 13%, 47% women) and University (*n* = 1961, ∼22 y.o., prior experience: 9%, 66% women) completed the survey. Among the students, the awareness of stroke general knowledge, associated risk factors, and warning signs and symptoms varied between 42%‒66%. When stimulated, less than 52% of the students associated stroke with hypercholesterolemia, smoking, diabetes, and hypertension. When stimulated, 62%‒65% of students recognized arm weakness, facial drooping, and speech difficulty; only fewer identified acute headache (43%). Interestingly, 67% knew the EMS number; 81% wanted to have stroke education at school, and ∼75% wanted it mandatory. Women, higher education, and prior experience were associated with higher scores of knowing risk factors (OR = 1.28, 95% CI: 1.10‒1.48; OR = 2.12, 95% CI: 1.87‒2.40; OR = 1.46, 95% CI: 1.16‒1.83; respectively), and warning signs- symptoms (OR = 2.22, 95% CI: 1.89‒2.60; OR = 3.30, 95% CI: 2.81‒3.87; OR = 2.04, 95% CI: 1.58‒2.63; respectively).

**Conclusion:**

Having higher education, prior experience, and being a woman increases stroke-associated risk factors, and warning signs and symptoms identification. Schoolchildren and adolescents should be the main target population for stroke awareness.

## Highlights

• Higher education, prior experience, and being women improved the odds of identifying stroke warning signs and symptoms as associated risk factors
• Improving knowledge, skills, and attitude on acute stroke in the school community may represent a significant advance in public health management
• Future stroke awareness campaigns and educational efforts should focus on schoolchildren and adolescents, especially in low-income countries

## Introduction

The increasing global burden of stroke is a leading cause of death and disability [1]. In most cases, stroke is preventable, and thus primary prevention is one of the foremost health priorities worldwide. According to previous reports, in 2017, approximately 460,000 people in Europe [2], 146,383 in the United States [3], and over 260,000 in Latin America died of a stroke [4]. The incidence and mortality rates have decreased in recent decades. However, the absolute numbers of new and recurrent stroke cases have increased, mainly due to an unhealthy lifestyle, increased cardiovascular risk factors throughout life, and longer life expectancy [4].

Recognition of acute stroke Warning Signs and Symptoms (WSS), awareness of the Associated Risk Factors (ARF), and the early activation of Emergency Medical Service (EMS) are critical factors for reducing the delay between the clinical onset and hospital arrival, improving medical treatment and patient outcomes, preventing new stroke cases and reducing mortality and disability. In 2019, more than 48,236 deaths were related to stroke in Brazil [5]. KIDS SAVE LIVES Brazil (KSLB) is a new program that teaches children, adolescents, and adults of the public school communities about basic concepts in the prevention, recognition, and first actions to take in cases of stroke, sudden cardiac arrest, and choking in babies, children, and adults [6]. By promoting health education for young people, the program has a significant effect, as it spreads knowledge from the school members to families and neighbors. However, little is known about people's awareness of stroke, ARF, and WSS in Brazilian school communities. Thus, a survey of students’ knowledge of stroke and understanding of their attitude gaps would be helpful to develop adequate educational approaches for use in different settings.

This study aimed to investigate students' general knowledge of stroke, ARF, WSS (facial droop, arm weakness, and speech difficulties), and EMS phone numbers at three educational stages (elementary/middle school, high school, and university) in Brazilian schools.

## Methods

This observational cross-sectional study, which was conducted from March 2019 to December 2020, was approved by the Ethics Committee of the University of São Paulo Medical School (CAAE 25218819.0.0000.0065 and 05564819.1.0000.0065). The authors invited students aged 7-years and older of both sexes from three public schools: Professora Elza Saraiva Monteiro State School, Professora Antonieta Borges Alves State School, and the University of São Paulo. These three schools representing different educational stages in the city of São Paulo, Brazil, were selected by convenience. Students aged ≥ 18 years signed the informed consent form, while those aged < 18 years gave permission accompanied by their parents’ or legal guardian's consent.

The survey was administered in paper-and-pencil format to the elementary/middle and high school students and via a web application to the University of São Paulo undergraduates. The survey comprised 16 multiple-choice questions. The three domains included four questions each (Table 1) on (1) general knowledge of stroke, (2) ARF, and (3) WSS. The score for each domain ranged from 0 to 10 points. The authors included an additional yes/no question on previous experience of witnessing a stroke in a family member or neighbor/friend or having contact with a stroke victim in the family or neighborhood. The authors also included two yes/no questions on receptiveness to learning about stroke at school and whether it should be a required topic. For the range of 0 to 10 points, the authors arbitrarily set ≤ 5 points as a lower grade and > 5 points as a higher grade. Last, the authors included a yes/no question on chest pain and coughing as distractors. The questionnaire is available in the supplementary material.

**Table 1:** Frequencies (n, percentage) of the students’ answers on general knowledge of stroke, associated risk factors, and warning signs and symptoms according to the educational stages, analyzed with the Chi-Square test.

Knowledge domains		Groups *n* (%)		
	Elementary/Middle school	High School	University	*p*-value
**General knowledge of stroke**				
Where does the stroke take place? (Brain)	665 (56.0)	625 (77.5)	1793 (91.4)	<0.001
Is there a risk factor associated with stroke (Yes)	579 (48.8)	311 (38.6)	1642 (83.7)	<0.001
Are there warning signs and symptoms (Yes)	485 (40.9)	264 (32.8)	1126 (57.4)	<0.001
What is the EMS phone number (192)	751 (63.3)	610 (75.7)	1286 (65.6)	<0.001
**Associated risk factors**				
Hypertension (Yes)	217 (18.3)	264 (32.8)	1566 (79.9)	<0.001
Diabetes (Yes)	263 (22.2)	216 (26.8)	712 (36.3)	<0.001
Dyslipidemia (Yes)	387 (32.6)	310 (38.5)	1354 (69.1)	<0.001
Smoking (Yes)	330 (27.8)	166 (20.6)	1075 (54.8)	<0.001
**Warning signs and symptoms**				
Facial droop (Yes)	392 (33.0)	409 (50.7)	1679 (85.6)	<0.001
Speech difficulties (Yes)	408 (34.4)	398 (49.4)	1761 89.8)	<0.001
Arm weakness (Yes)	382 (32.2)	382 (47.4)	1684 (85.9)	<0.001
Headache (Yes)	175 (14.7)	179 (22.2)	1340 (68.3)	<0.001

For the statistical analysis, the authors used SAS software, version 9.3, of SAS System for Windows, copyright 2011 (SAS Institute Inc., Cary, NC, USA). The sample calculation was conducted in a free program (www.select-statistics.co.uk). The main variable was WSS recognition, with 80% power, α = 0.05, a sample proportion of 66% in adults [7] and a sample proportion of 56% in schoolchildren [8], resulting in 370 subjects for each group. The authors presented descriptive data as absolute (%) and mean values (SD), when appropriate. The authors used the Chi-Square test to analyze categorical variables between elementary/middle school, high school, and university groups. A multiple logistic regression model was used to assess the three domains (general knowledge of stroke, ARF, and WSS) categorized into yes (score > 5) or no (score ≤ 5), adjusted for age, sex, and educational stage, as well as previous experience. The significance level was established as a p-value < 0.05.

## Results

Of the 2,993 students in elementary/middle and high school, 67% answered the questionnaire. Of the 65,342 undergraduates, 3% answered the questionnaire. The demographic characteristics of the 3,954 students who participated in the study are shown in Table 2. They were distributed into elementary/middle school (30.0%), high school (20.4%), and university (49.6%). This study also showed that 431 undergraduates were enrolled in health care programs (22%), while 1,530 students were enrolled in other programs. Among the students, women were more prevalent (*p* < 0.001) in the university group (66.1%) than in the elementary/middle school (51.1%) and high school groups (47.3%).

**Table 2:** Students’ demographic characteristics (*n* = 3,954) according to educational stages.

Educational Stage	Grade	n	Men (%)	Mean age (SD)	Min‒Max
Elementary/middle School	2	22	59.1	7.6 (0.5)	7‒8
	3	30	63.3	8.7 (0.5)	8‒9
	4	39	43.6	9.9 (0.4)	9‒11
	5	56	54.4	10.9 (0.9)	10‒14
	6	208	51.0	11.8 (0.6)	11‒16
	7	297	49.2	12.9 (0.7)	12‒16
	8	304	44.4	13.9 (0.7)	13‒17
	9	231	48.9	14.9 (0.8)	14‒18
**Total**		**1187**	**48.9**	**13.0 (1.8)**	**7‒18**
High school	10	282	52.5	15.8 (0.9)	15‒19
	11	228	54.4	16.7 (0.8)	15‒22
	12	296	51.7	17.3 (0.8)	15‒20
**Total**		**806**	**52.7**	**16.6 (1.1)**	**15‒22**
University	13	523	40.5	20.0 (4.9)	16‒57
	14	435	31.3	21.0 (4.7)	17‒59
	15	362	30.4	23.0 (5.6)	19‒69
	16	320	34.7	24.0 (6.3)	19‒65
	17	228	31.1	25.0 (5.0)	21‒61
	18	93	26.9	25.8 (5.1)	21‒49
**Total**		**1961**	**33.9**	**22.3 (5.6)**	**16‒69**

A significant number of undergraduates correctly answered the questions in the three domains, more than participants in the lower educational stages. On the other hand, they recorded a higher proportion (26.1%) of wrong answers on whether a stroke is related to chest pain than the elementary/middle school and high school students (16.1% and 20.5%, respectively, *p* < 0.001). In general, the elementary/middle school students recorded lower proportions of correct answers in the three domains. They also associated stroke with coughing more frequently (6.6%) than the high school students and undergraduates (4.3% and 4.8%, respectively, *p* = 0.042).

The authors detected more frequent previous experiences (*p* < 0.001) in students attending elementary/middle school (13.5%) and high school (12.5%) than in undergraduates (8.9%).

Of a total of 3,954 students, 80.7% were willing to learn about stroke at school, and 75.2% reported that it should be a required topic at school. Among them, undergraduates (85.8%) were more willing (*p* < 0.001) to have stroke included as an educational topic at school and 83.6% to have it included as a required topic than elementary/middle school (74.7% and 64.5%, respectively) and high school students (77.1% and 70.5%, respectively).

The multivariate logistic regression model (Table 3) was adjusted for age, sex, educational stage, and previous experience in the knowledge of stroke domains. Higher education was a significant independent factor for all domains. Sex, educational level, and previous experience were independent variables for the recognition of ARF and WSS.

**Table 3:** Odds ratio of the three domains (General Knowledge of Stroke, Associated Risk Factors and Warning Signs and Symptoms) categorized into yes (score > 5) or no (score ≤ 5) according to age, sex, educational stage, and previous experience, using multivariate logistic regression analysis with 3,954 observations.

Domains	Variables	OR	p-value	95% CI
General knowledge of stroke				
	Age	1.04	<0.001	1.02 – 1.06
	Women	1.11	0.147	0.97 – 1.27
	Educational stage	1.86	<0.001	1.65 – 2.09
	Previous experience	0.98	0.817	0.79 – 1.21
Associated risk factors				
	Age	1.06	<0.001	1.04 – 1.08
	Women	1.28	0.001	1.10 – 1.48
	Educational stage	2.12	<0.001	1.87 – 2.40
	Previous experience	1.46	0.001	1.16 – 1.83
Warning signs and symptoms				
	Age	1.10	<0.001	1.06 – 1.13
	Women	2.22	<0.001	1.89 – 2.60
	Educational stage	3.30	<0.001	2.81 – 3.87
	Previous experience	2.04	<0.001	1.58 – 2.63

## Discussion

The authors provided an updated overview of students' knowledge of stroke in different educational stages in Brazilian public schools, specifically encompassing schoolchildren, adolescents, and young adults. The authors identified misconceptions and educational challenges that may help outline more efficient educational strategies for this young population. A higher educational level, previous experience, and being a woman are independent factors for achieving higher scores for general knowledge of stroke, associated risk factors, and recognition of warning signs and symptoms. Most students reported that knowledge of stroke is a crucial topic to be taught at school and declared that it should be required. The fields of study varied among undergraduates – approximately 25% of them were enrolled in biological and health fields, whereas the remaining respondents were enrolled in programs for other fields.

Public campaigns and educational efforts have been conducted worldwide to increase knowledge of stroke, as it persists as one of the major causes of disability, decreased quality of life, and death. However, the lack of knowledge of stroke ARF and WSS is still a major global challenge. The authors compare several related studies in Table 4 for a brief review 7, 8, 9, 10, 11, 12, 13, 14, 15, 16, 17, 18, 19, 20, 21, 22, 23. Schoolchildren and adolescents are an interesting target population for health education [4,6,11,19,20]. They can take leading roles, shaping their choices and attitudes toward a healthy lifestyle. They can also cooperate, sharing knowledge of stroke with parents, friends, and neighbors to prevent future risk factors and improve health outcomes [6,19,20]. In the present study, the majority of the students, particularly the undergraduates, knew that the brain is the part of the body affected by a stroke, similar to other investigations conducted worldwide (Table 4) [8,10,12,15,16,23]. The average recognition of ARF for hypertension (∼18 and ∼33%, respectively), diabetes (∼22 and ∼27%, respectively), smoking (∼28 and ∼21%, respectively), and dyslipidemia (∼33 and 39%, respectively) in schoolchildren and adolescents was similar to other studies [8,16]. However, the present results from schoolchildren and adolescents were alarmingly poor compared with other countries [11,15,20]. The high proportion of undergraduates who identified ARF in the present study was comparable with studies performed with samples of the general public [10,12, 13, 14,16,17].

**Table 4:** Worldwide lay knowledge of stroke in chronological order of appearance in the literature.

Authors	Subjects	Study design assessments	Baseline knowledge of stroke			Conclusions
			Associated Risk Factors (ARF)	Warning Signs and Symptoms (WSS)	Emergency Medical Services (EMS)	
Pontes-Neto et al., 2008 [9] Brazil	*n* = 801	Cross-sectional	None: ∼19%	None: ∼22%	EMS phone number: ∼35%	Alarming lack of knowledge on EMS activation and patient treatment
	General public	Interview survey	Hypertension: ∼30%		EMS activation at onset: ∼51% Willingness to go to the emergency department by own means: ∼40%	
	Mean age: ∼39 y.o. (range: 18‒80 y.o.)		Diabetes: ∼2%			
			Smoking: ∼50%			
	∼54% of women		Dyslipidemia: ∼15%			
			Obesity: ∼5%			
			Alcohol consumption: ∼21%			
Robinson et al., 2013 [10] United Kingdom	*n* = 1300	Cross-sectional	Hypertension: ∼90%	Facial droop: ∼89%	Brain (site of stroke): ∼68%	The FAST campaign was effective for raising public stroke awareness
	General public		Diabetes: ∼51%	Arm weakness: ∼83%		
	Mean age: ∼49 y.o. (range: 10‒87 y.o.)	Interview survey	Smoking: ∼74%	Slurred speech: ∼91%		
			Alcohol consumption: ∼54%			
	∼61% of women					
Panicio et al., 2014 [7] Brazil	*n* = 104	Cross-sectional	Not provided	Recognition: ∼66%	Not provided	Lack of knowledge about the therapeutic time window may affect early hospital arrival despite WSS knowledge
	Acute stroke patients and their families					
	Mean age: ∼64 y.o.	Interview survey				
	∼45% of women					
Shigehatake et al., 2014 [11] Japan	*n* = 493	Cohort	Hypertension: ∼76%	Facial droop: ∼33%	EMS activation at onset: ∼85%	Manga and other educational materials can improve students’ stroke knowledge
		Questionnaires	Hyperglycemia: ∼52%	Hemiparesis: ∼52%		
			Smoking: ∼54%	Slurred speech: ∼60%		
	Students (10^th^ grade) (range 12‒13 y.o.)		Dyslipidemia: ∼55%	Sudden headache: **∼**55%		
			Obesity: ∼22%			
			Alcohol consumption: ∼72%			
Yang et al., 2014 [21] China	*n* = 1101	Cross-sectional	Not provided	None: ∼13%	EMS activation at onset: ∼28‒43%	Low stroke awareness and response at the onset, particularly by the lower education level and older people
	General public			Facial or limb weakness: ∼76%		
	Mean age: ∼58 y.o. (range: 18‒91 y.o.)	Interview survey				
				Slurred speech: ∼69%		
	∼62% of women			Sudden headache: ∼36%		
Ntaios G et al., 2015 [13] Greece	*n* = 723	Cross-sectional	Hypertension: ∼66%	Facial droop: ∼38%	EMS activation at onset or willingness to go to the emergency department by own means: ∼69%	Stroke knowledge is moderately poor in general, and it is important to develop new educational strategies to improve stroke risk perception
	Mean age ∼47 y.o.		Smoking: ∼44%	Arm or leg paralysis: ∼49%		
	General public	Interview survey (by phone)	Obesity: ∼34%			
	∼58% of women			Slurred speech: ∼44%		
Matsuzono et al., 2015 [20] Japan	*n* = 2040	Cohort	Students and their parents:	Students and their parents:	EMS activation at onset: ∼61% and ∼83%	Manga and other educational materials can improve students’ stroke knowledge, and their parents also learn through the children's communication of what they learned
	Students (10^th^‒12^th^ grades) and their parents	Questionnaires	Hypertension: ∼82% and ∼91%	Facial droop: ∼45% and ∼71%		
			Diabetes: ∼57% and ∼46%	Arm weakness: ∼58% and ∼73%		
			Smoking: ∼64% and ∼82%	Slurred speech: ∼63% and ∼91%		
			Dyslipidemia: ∼58% and ∼73%	Sudden headache: ∼68% and ∼81%		
			Overweight: ∼28% and ∼51%			
Ramírez‑Moreno et al., 2015 [23] Spain	*n* = 2409	Cross-sectional	Men and women:	Men and women:	Men and women:	Lack of knowledge in the general public. Women perform better in general.
	General Public					
	Mean age: ∼66 y.o.		Hypertension: ∼33% and ∼41%	Paralysis or weakness: ∼32 and ∼31%	EMS activation at onset or willingness to go to the emergency department by own means: ∼45 and 39%	
	∼60% of women					
		Interview survey	Diabetes: ∼13% and ∼15%	Slurred speech: ∼11% and ∼13%		
			Smoking: ∼54% and ∼48%	Sudden headache: ∼30% and ∼31%		
			Dyslipidemia: ∼25% both		Brain (site of stroke): ∼71 and 72%	
			Obesity: ∼15% and ∼13%			
			Alcohol consumption: ∼48% and ∼40%			
Kilkenny et al., 2016 [14] Australia	*n* = 591	Cohort	Hypertension: ∼89%	Facial droop: ∼86%	EMS activation if observed facial droop: ∼92%	Educational programs towards stroke awareness can effectively improve the general public knowledge of stroke
	General Public	Questionnaires	Diabetes: ∼36%	Arm weakness: ∼81%		
	∼68% of women		Smoking: ∼62%	Slurred speech: ∼91%		
			Dyslipidemia: ∼58%			
			Alcohol consumption: ∼27%			
Park et al., 2017 [15] Korea	*n* = 1052	Cohort	Hypertension: ∼57%	Arm or leg paralysis: ∼26%	EMS activation at onset: ∼80%	Associated risk factors and warning signs and symptoms are important areas to address
	Students (11‒12^th^ grade)	Questionnaires	Diabetes: ∼36%			
			Smoking: ∼49%	Sudden headache: ∼6%	Brain (site of stroke): ∼74%	
			Dyslipidemia: ∼63%			
			Obesity: ∼51%			
			Alcohol consumption: ∼51%			
Marto et al., 2017 [16] Portugal	*n* = 1108	Randomized trial	Students and their parents:	Students and their parents:	Students and their parents:	Lectures at school for students and their parents increased stroke knowledge
	Students (8^th^ grade) and their parents	Questionnaires	Hypertension: ∼63% and ∼80%	Facial droop: ∼25% and ∼63%	EMS activation at onset: ∼45% and ∼56%	
	Mean age: ∼23 y.o.		Diabetes: ∼21% and ∼29%	Arm weakness: ∼40% and ∼65%		
			Smoking: ∼37% and ∼61%	Slurred speech: ∼40% and ∼65%	Brain (site of stroke): ∼84% and ∼75%	
			Dyslipidemia: ∼53% and ∼77%			
			Obesity: ∼39% and ∼76%			
			Alcohol consumption: ∼37% both			
Umar et al., 2019 [8] United States	*n* = 603	Cross-sectional	Recognition: ∼11%	FAST acronym: ∼56%	EMS phone number: ∼65%	Mothers´ educational level and race were associated with greater stroke knowledge with an alarming lack of knowledge on risk factors and symptoms at middle and high school
	Students (7^th^‒12^th^ grade)					
	Mean age: ∼15 y.o. (range: 12‒18 y.o.)	Paper-and-pencil survey			Brain (site of stroke): ∼50%	
	∼50% of women					
Dossi et al., 2019 [17] Argentina	*n* = 12,710	Cross-sectional	Hypertension: ∼69%	Recognition: ∼73%	EMS activation at onset: ∼26%	Stroke knowledge is moderate with poor-risk perception, requiring educational programs
	General public	Household survey	Dyslipidemia: ∼69%	Sudden headache: ∼66%		
	Mean age: ∼51 y.o.		Stroke is preventable: ∼67%		Willingness to go to the emergency department by own means: ∼52%	
	∼69% of women		Lifestyle measures are useful: ∼58%			
Houessou et al., 2021 [18] Benin	*n* = 4671	Cross-sectional	None: ∼91%	None: ∼95%	Not provided	Age, educational level, and prior experience were associated with stroke knowledge
	General public					
	Mean age: ∼28 y.o. (range: 15‒99 y.o.)	Household survey				
	∼51% of women					
Calderaro et al., 2021 [current] Brazil	*n* = 3954	Cross-sectional	(a) Elementary/Middle School:	(a) Elementary/Middle School	(a) Elementary/Middle School:	Having a higher educational level, being female, and having prior experience increase the identification of associated risk factors and warning signs and symptoms. Stroke awareness should be emphasized among schoolchildren and adolescents.
	Students (2^nd^ to 18^th^ grade)	Paper-and-pencil and online survey	None: ∼51%	None: ∼46%	EMS phone number: ∼63%	
	Mean age: ∼18 y.o. (range: 7‒69 y.o.)		Hypertension: ∼18%	Facial droop: ∼33%		
	∼58% of women		Dyslipidemia: ∼33%	Arm weakness: ∼32%	Brain (site of stroke): ∼56%	
			Diabetes: ∼22%	Slurred speech: ∼34%		
			Smoking: ∼28%	Sudden headache: ∼15%		
			(b) High School:	(b) High School:	(b) High School:	
			None: ∼46%	None: ∼32%	EMS phone number: ∼76%	
			Hypertension: ∼33%	Facial droop: ∼51%		
			Dyslipidemia: ∼39%	Arm weakness: ∼47%	Brain (site of stroke): ∼78%	
			Diabetes: ∼27%	Slurred speech: ∼49%		
			Smoking: ∼21%	Sudden headache: ∼22%		
			(c) University:	(c) University:	(c) University:	
			None: ∼14%	None: ∼6%		
			Hypertension: ∼80%	Facial droop: ∼86%	EMS phone number: ∼66%	
			Dyslipidemia: ∼69%	Arm weakness: ∼86%		
			Diabetes: ∼36%	Slurred speech: ∼90%	EMS phone number: ∼66%	
			Smoking: ∼55%	Sudden headache: ∼68%		

Stroke WSSs have been included in awareness campaigns and educational materials using the acronym FAST [6,10], an easy, practical, and straightforward learning strategy to recall facial droop, arm weakness, slurred speech, and time urgency. Unfortunately, a very low percentage (∼5%) of the general public is aware of stroke WSS in some countries [18]. To the best of our knowledge, this study is the first to present the rates of Brazilian students who had absolutely no knowledge of stroke WSS in public elementary/middle school (∼46%) and high school (∼32%). Interestingly, the percentage of adults with no knowledge of all stroke WSS decreased significantly in recent decades, from ∼22% in 2008 [9] and ∼34% in 2014 [7] to ∼6% in the present study (2021). However, the adults who participated in the present study were undergraduates from the University of São Paulo, who might have had different backgrounds from those of respondents of previous Brazilian studies that were performed with the general public [6] or with patients who experienced a stroke [7].

Surprisingly, most students from the three educational levels knew that stroke occurs in the brain. However, a lower proportion of them recognized sudden headaches as one of the possible stroke WSSs. The authors suggested that the general public may be unaware that an acute and uncommon headache may be associated with stroke, a widespread medical emergency event 2, 3, 4, 5. Additionally, the general public may confuse ischemic heart diseases with stroke [22,23]. Higher proportions of undergraduates positively identified each of the four WSSs listed in the present study. The scores of knowledge of stroke WSS increased with a higher educational stage (3.3-fold) and previous experiences with stroke in family or neighbors (2-fold), which may be related to other studies [12,18]. The authors found that previous experience (i.e., previous contact with a stroke victim in the family or the neighborhood) increases the chance of having more knowledge about stroke WSS and ARF, possibly because the students may want to know more about stroke by discussing it with peers (family, friends, and colleagues), researching it on the internet, learning in courses, and other sources of information. The authors also determined the role of sex, and women exhibited greater knowledge of WSS (2.2-fold) and ARF (1.3-fold). The authors postulated that women may be more interested and better informed about health conditions, prevention, and treatment of common diseases [23,24].

According to this study, a large proportion of students from elementary/middle school (∼63%), high school (∼76%), and university (66%) correctly identified the EMS phone number (192 in Brazil) among four other phone numbers. In a study from 2008, only 35% of the general public knew the valid phone number, which paralleled the willingness to call EMS at stroke onset [9]. Due to effective public campaigns for stroke awareness, including the national EMS, the proportion of students knowing the correct phone number approximately doubled in Brazil over the last two decades. These results are consistent with other studies assessing middle and high school students and the general public [8,12,13,16,17]. However, these results are still lower than those of other studies assessing students and the general public from developed countries [11,14,15]. In this sense, knowing the correct EMS phone number is a crucial aspect on which to focus to ensure that the willingness to call EMS transforms into action, saving time and lives. The role of the EMS is to safely and quickly transport victims to an adequate health care center. Hence, it is a critical component of the stroke survival chain, along with correct diagnosis and proper treatment to improve the quality of life. However, the recognition of WSS was not associated with early hospital arrival, indicating that a lack of knowledge of the therapeutic time window for adequate stroke diagnosis and treatment persists.

The present study has limitations. Students were recruited only from public schools, despite the significant quality mismatches between private and public schools in Brazil, as private schools usually provide higher quality education. In this regard, the University of São Paulo is the most prestigious higher education institution in Latin America, which might have provided a sample of undergraduates with a higher literacy level and socioeconomic status. However, approximately 50% of the undergraduates at the University of São Paulo come from public high schools, which may help balance this selection. Another limitation was that the authors used a closed-ended survey questionnaire, which may have helped students fill in the blanks. One might argue that the answers may have been systematic. However, the undergraduates’ proportion of wrong answers was only 5%‒10% higher than those of participants at lower educational levels when considering “whether a stroke is related to chest pain”. In addition, the authors used the same closed-ended questionnaire for all students in either a paper-and-pencil format or online forms, which may reduce this possible inconsistency. Students first answered questions on general knowledge (e.g., “Is there any risk factor associated with stroke?”), and the authors subsequently stimulated the recognition of hypertension, diabetes, dyslipidemia, and smoking using a specific question for each of the four main ARFs.

The present study showed that the average knowledge of stroke was particularly deficient in students in the lower educational levels in public schools. In general, most of these students are from families with low incomes and socioeconomic status. In addition, the present study showed that students from lower educational levels had more previous experiences with stroke in the family or neighborhood than undergraduates. These results are consistent with other studies [25] showing substantial socioeconomic inequalities in Brazil, with a high prevalence of chronic diseases (such as stroke and other cardiovascular disorders and associated disabilities) in the lower socioeconomic classes. Other studies also note that younger age groups, especially in low- and middle-income countries, are at risk of stroke, possibly due to an unhealthy lifestyle, causing significant economic and quality-of-life losses 26, 27, 28. The Brazilian population is composed of individuals with different socio-cultural backgrounds and racial diversity. The health care system is based on the Family Health Strategy with a community perspective [29] for health promotion and prevention and treatment of diabetes, hypertension, and other cerebrovascular and cardiovascular diseases [30]. In this sense, the present study highlights that the poor knowledge of these important risk factors continues to be an alarming health care problem, despite widespread access to family medicine [29].

In this context, schoolchildren, adolescents, and young adults are potential multipliers 31, 32 of knowledge of stroke and skills to perform first actions outside the hospital setting. Improving schoolchildren and adolescents’ knowledge, skills, and attitudes regarding acute stroke may represent a significant advance in public health management. Future stroke awareness campaigns and educational efforts should focus on schoolchildren and adolescents, especially from countries with lower incomes.

In conclusion, higher education is an independent factor contributing to all domains of stroke knowledge. Having previous experiences and being a woman increase the identification of associated risk factors and warning signs and symptoms.

## Funding

The authors would like to thank São Paulo State Research Foundation (FAPESP 2019/27652-4), Pró-Reitoria de Graduação (Programa Aprender na Comunidade), and University of São Paulo Rectory (Santander USP Municípios) for the scholarships to undergraduates.

## Disclosures

Federico Semeraro is Chair-Elect and SEC BLS co-chair of the European Resuscitation Council; ILCOR BLS Task Force Member. Maria José C. Carmona receives fees from Cristália Pharma Ind., Medtronic PLC, and União Química Pharma S.A. Editor of the Brazilian Journal of Anaesthesiology. Bernd W. Böttiger is the treasurer of the European Resuscitation Council (ERC); Chairman of the German Resuscitation Council (GRC); Member of the Advanced Life Support (ALS) Task Force of the International Liaison Committee on Resuscitation (ILCOR); Member of the Executive Committee of the German Interdisciplinary Association for Intensive Care and Emergency Medicine (DIVI), Founder of the German Resuscitation Foundation, Co-Editor of “Resuscitation”; Editor of the Journal “Notfall + Rettungsmedizin”, Co-Editor of the Brazilian Journal of Anaesthesiology. He received fees for lectures from the following companies: Forum für medizinische Fortbildung (FomF), Baxalta Deutschland GmbH, ZOLL Medical Deutschland GmbH, C.R. Bard GmbH, GS Elektromedizinische Geräte G. Stemple GmbH, Novartis Pharma GmbH, Philips GmbH Market DACH, Bioscience Valuation BSV GmbH. Naomi K. Nakagawa is the National Coordinator of Kids Save Lives Brazil. Co-Editor of Clinics. Member of the Basic Life Support Science and Education Committee of the European Resuscitation Council. The other authors have no conflict of interest to declare.

## Conflicts of interest

The authors declare no conflicts of interest.

## Appendix Supplementary materials

Image, application 1
