# Supplementary material for: The lack of knowledge on acute stroke in Brazil: A cross-sectional study with children, adolescents, and adults from public schools
Source: Clinics (Sao Paulo). 2022 Jun 28;77:100052. doi: 10.1016/j.clinsp.2022.100052 (PMC9253714; doi:10.1016/j.clinsp.2022.100052)
Supplement: Supplementary file 1 [file mmc1.docx]

CLINICS-D-22-00137_Supplementary Material

**Supplementary**

| **KIDS SAVE LIVES BRAZIL** |
| --- |
| **Stroke Questionaire** |
| Full name: |
| Gender: |
| School level: |
| Grade: |
| 1. What is the Brazilian emergency phone number? |
| 2. In what part of our body a stroke happens? |
| 3. Does stroke present with signs and symptoms? |
| 4. Are there risk factors for stroke? |
| 5. Is hypertension a risk factor for stroke? |
| 6. Is diabetes a risk factor for stroke? |
| 7. Is dyslipidemia a risk factor for stroke? |
| 8. Is smoking a risk factor for stroke? |
| 9. Is facial drooping a sign of stroke? |
| 10. Is chest pain a symptom of stroke? |
| 11. Is coughing a sign of stroke |
| 12. Is trouble speaking a sign of stroke? |
| 13. Is headache a symptom of stroke? |
| 14. Is trouble walking a risk of stroke? |
| 15. Do you know what to do when you witness someone having a stroke? |
| 16. Have you ever witnessed a stroke? |
| 17. Would you like to have education on stroke in your school? |
| 18. In your opinion, should education on stroke be mandatory in schools? |
